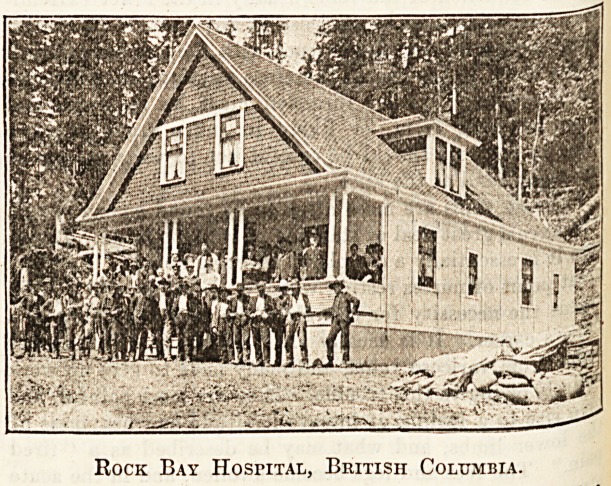# "The Hospital" Nursing Section

**Published:** 1906-07-21

**Authors:** 


The Hospital.

Nursing Section.

Contributions for "THE HOSPITAL," should be addressed to the EDITOR, "THE HOSPITAL" NURSING SECTION, 28 & 29 Southampton Street, Strand, London, W.C.

No. 1 03;—Vol. XL. SATURDAY, JULY 21, 1906.

### Notes on News from the Nursing World.

### THE EDUCATION BILL AND QUEEN'S NURSES.

The President of the Board of Education, speaking in the House of Commons last Monday on a proposal by Mr. Tennant for the compulsory medical inspection of children attending the elementary schools, which was supported by Sir William Collins and others, said he questioned whether the work could be undertaken by medical officers of health, whose hands were already full; but he thought that Queen's nurses and district nurses might render valuable assistance without it being necessary to engage their entire services. He suggested that they could treat ailments which were not serious, follow the little patients home, and tell their mothers what was the matter with them. This view appeared to commend itself to Parliament, and in the event of the Bill becoming law, we may anticipate the partial employment, by the local authorities at any rate, of qualified nurses under its provisions. In that case we trust great care will be taken to guard against the possibility of their assumption of obligations which can only be satisfactorily performed by medical men.

### NURSING THE INJURED AT HANDCROSS.

The disastrous motor accident at Handcross Hill last week tested, to the utmost extent, the capacity and resourcefulness of all in the neighbourhood. It was between eleven and twelve o'clock on Thursday morning that the Honorary Secretary of Crawley Cottage Hospital reported the event to the matron, and asked her to go to the scene of the accident. Thanks to the good offices of one of the ladies of Crawley, Miss Heriot, taking with her a few dressings, was at once able to proceed to Handcross, four miles distant, by motor. On arriving at the Red Lion Hotel she first attended two men, one with a badly cut head, and the other not very much hurt. The large club-room presented a terrible sight. Here, as fast as the injured arrived, they were placed on mattresses on the floor and seen to, dressings and splints, or whatever was wanted, being obtained without delay from the Crawley Hospital. The matron took the first man, who was suffering from a bad scalp wound and a fractured arm, back by motor to the hospital; and by the time she had got him to bed three more patients arrived. About three o'clock in the afternoon three nurses, including the casualty sister, arrived from the Sussex County Hospital, Brighton; owing to the energy of the workers who were able to be on the scene earlier, they found the arrangements at the hotel well forward. Two large rooms had been converted into wards of four beds each; and in a small corridor, curtained off, another temporary resting-place was established. Later on two men were conveyed to the Sussex County Hospital under the care of the sister; the two nurses from Brighton were left in charge of the patients at the Red Lion; and in the evening two more men were taken by motor omnibus to Crawley Cottage Hospital. As the latter building only contains six beds, the matron had, of course, to improvise a seventh. The nursing staff is limited to one, in addition to the matron, but their labours were materially lightened by the kindness of the medical men and the willingness of the people at the hotel and elsewhere to render assistance. The patients also helped the nurses by the pluck and the patience which they manifested. At the same time, great credit must be given to all concerned for the success with which they rose to the occasion.

### ECONOMY AT THE EXPENSE OF EFFICIENCY.

The question of holidays has just been engaging the attention of two Boards of Guardians. In one case the Guardians responsible for the management of the Sick Asylum at Hendon have cut down the holidays of the nurses from three weeks to a fortnight, and that of the matron from a month to three weeks.. This has been done in the avowed interests of economy. At Dudley a proposal was made, also on the ground of economy, to abandon the custom of engaging a special nurse to fill the places of those away on holiday, and to compel a diminished staff to perform the duties. The proposal was wisely rejected. Under no circumstances whatever should there be an inadequate nursing staff. The fact that nurses are on holiday is not a sufficient reason why the patients in an institution should not be properly cared for. With regard to the determination of the Middlesex Guardians, we do not think that the ratepayers desire that a small saving should be effected by the means adopted. A holiday of three weeks is, in our judgment, requisite for a nurse who works hard all the rest of the year; and even apart from this consideration it is not fair to break any rule on the faith of which the nurses undertake to serve the Guardians.

### DISOBEDIENT AND INCAPABLE MIDWIVES.

At the last meeting of the Central Midwives Board no fewer than 18 women were charged with breaking the rules. One of the worst cases was that of a person in London, who was convicted of having on two different occasions employed her husband, an unqualified individual, to discharge the duties of a midwife, for which she had been employed; while on a third occasion she had called him in instead of a medical practitioner. Another woman, in Norfolk, who had previously been suspended, was proved to have attended several confinements while she was under suspension, and to have persistently disobeyed the rules of the Board. In a third case, in Yorkshire, the death of one, and the serious illness of another patient had arisen through the disobedience of the midwife; and in a fourth a woman was convicted of drunkenness as well as neglect of the rules. The names of all these were, of course, removed from the roll, and also those of eight others, most of the remainder of the women charged being either censured or cautioned. The Board may be open to criticism in some respects for its decisions, but it seems to discharge its obligations in hearing and disposing of the cases of disobedient, incapable, and dangerous midwives in quite a judicial spirit.

### OVERWORK AT ASTON.

THE Aston Guardians hasten slowly. As we intimated a fortnight ago, they rejected the proposal to appoint six additional probationers at the infirmary in favour of an amendment reducing the increase to three, notwithstanding the strong reasons given by the Infirmary Committee for advising the appointment of the larger number. At their last meeting a suggestion was made that the Infirmary Committee should invite an independent inquiry into the staffing of the infirmary, and it was warmly welcomed by the Chairman of the Committee. In spite of the approval of the Chairman of the Board, or perhaps because he said that he believed the result of the investigation would be a recommendation that six nurses should be appointed, a decision in the matter was deferred to the next meeting. This delay is the more astonishing in view of the statement of the Chairman of the Infirmary Committee "that only a week ago a consumptive patient was suffering acutely, and the nurse, having to go to another patient who was seriously ill, the former died before she returned." A fact like this, coupled with the fact that night nurses often have to go without food during the whole night owing to the pressure of work, should, at all events, be held sufficient to establish the need of an independent inquiry forthwith.

### A MOVEMENT TO LOWER THE STANDARD.

IT appears that the anxiety of the Swindon and Highworth Guardians to reduce the minimum age limit of probationers employed in Poor-law Institutions from twenty-one to eighteen, to which we recently referred, is receiving some support from other bodies who find it either difficult to secure, or to retain, a sufficient staff of nurses. We cannot, however, believe for a moment that in order to afford temporary relief to the authorities of a small minority of workhouse infirmaries the Local Government Board will sanction a step that would inflict a distinct injury on the increasingly numerous efficient training schools which are accustomed to look to Whitehall for protection and encouragement. It is not by admitting a brigade of immature girls into the wards that the troubles which are frequently reported in various parts of the country can be got rid of, but by satisfying the reasonable claims of the nursing staff to consideration in such matters as off-duty time, accommodation, and diet. The Local Government Board must be perfectly well aware that the supply of suitable probationers at the Poor-law Institutions conducted on sound principles, maintaining a high standard of nursing, is in excess of the demand. The reduction of the minimum age to eighteen would, at the best, do more harm than good.

### IMPERIAL MILITARY NURSING SERVICE.

WE are officially informed that Miss J. H. Clay, sister in Queen Alexandra's Imperial Military Nursing Service has been transferred to the Royal Arsenal Hospital, Woolwich, from the Military Hospital, Portsmouth; and that Miss C. Winzer, staff nurse, has been appointed to the Military Hospital, Gosport. The appointment of Miss J. A. Howe as staff nurse has been confirmed, and the following staff nurses have been promoted to be sisters:—Miss G. M. Allen, Miss N. Blew, Miss K. M. Bulman, Miss L. M. Dann, Miss E. M. Fairchild, Miss E. Foster, Miss M. E. M. Grierson, Miss M. S. Ram, and Miss M. F. Steele.

### THE LATE MR. EDWARD RAWLINGS.

BY the death of Mr. Edward Rawlings the Royal National Pension Fund for Nurses and all associated with it lose much. Mr. Rawlings was one of the original members of the Council of the Fund; he devoted a very large amount of his time to the details of the work, and it is probable that on the average he attended more meetings of the Council than perhaps any other member. He was one of the most courteous, considerate, and kindly men that have ever lived, and he endeared himself to his colleagues, not only by his devotion to the work, but also by the acumen and generosity which always characterised his actions. Mr. Rawlings has left £500 to the Donation Bonus Fund, and his memory will long be cherished by all who had the privilege of knowing him intimately. We desire to offer to Mrs. Rawlings the sincerest condolences and sympathy, not only of the Council, but of all policy-holders connected with the Royal National Pension Fund for Nurses.

### THE INDIAN NURSING ASSOCIATION.

THE Up-Country Nursing Association, which, as our readers are well aware, has been doing its best under great financial difficulties, is to be absorbed in the Indian Nursing Association, which is being organised to provide skilled nurses throughout Northern India, by the Countess of Minto, wife of the Viceroy. Lady Minto, in an appeal from the Viceregal Lodge at Simla, states that the new system will furnish nurses not only in the United Provinces and the Punjab, as the Up-Country Nursing Association has hitherto done, but also in Burma, Eastern Bengal and Assam, the Central Provinces, the North-West Frontier Province, Baluchistan, Rajputana, and Central India, where at present there is no such organisation whatever. The fees for the services of the nurses will be arranged on a sliding scale in order that the Association may be the means of benefiting rich and poor alike. The scope of the work, however, depends materially upon the generosity of the public, and we trust that the new movement will obtain more substantial support than has been accorded to the Up-Country Association.

### GARDEN FETE FOR A BED AT GUY'S HOSPITAL.

On Wednesday last week a garden fête and fair was held in the grounds of "The Cedars," Belmont Hill, Lee, by the kind permission of Mrs. Penn, the object [illegible]ing to raise enough money to endow a bed in perpetuity in Guy's Hospital. The fête was opened by Mrs. H. Cosmo Bonsor who was supported by Mr. W. Arbuthnot Lane. The entertainment was splendidly organised, and every kind of amusement was provided. Stalls laden with pottery, silver, pictures, baskets, flowers, and fruit did good trade, and the side-shows were also well patronised, the "sick patient's" tent being a great favourite. The patient was an anæmic wax figure, who was carefully tucked up in an orthodox bed and tended assiduously by one of Guy's students and relays of nurses. Entertainments and concerts were given at intervals, and were a substantial source of income. Members of the nursing staff, and doctors and students also, were most energetic in their efforts to inveigle hesitating purchasers. The band of the Royal Artillery played during the afternoon.

### INTERESTING CEREMONY AT ST. MARY'S HOSPITAL.

The matron and all the sisters at St. Mary's Hospital, Paddington, who could be spared from the wards were present on Monday at the annual distribution in the library of prizes to the students of the Medical School by the Director-General of the Army Medical Service. The event of the afternoon was the unveiling of the memorial bronze erected in the entrance of the Clarence wing to the memory of members of the hospital and medical school who lost their lives during the South African war. These included a St. Mary's nurse, Sister Edith Manley Gardner, of the Army Nursing Service Reserve. After a speech by the chairman of the Board of Management, the ceremony itself was performed by the Director-General of the Army Medical Department. Tea was provided in the Board-room, and the Nurses' Home and wards of the hospital were open to visitors. The centre of attraction was the Crawshay ward, where the students' cot—for which an endowment is being raised by the students of the Medical School—was on view. The small occupant was not in the least disconcerted by the crowd of admirers. Another little man sat on his pillow playing with his toes and peeping out between times at the strangers. Not a single child out of the 12 cried, and all seemed quite happy and contented.

### SUSSEX COUNTY NURSING ASSOCIATION.

On Thursday last week the nurses working in districts in East Sussex affiliated to the Sussex County Nursing Association had their second annual gathering. The Marquis of Abergavenny invited them to Eridge Castle. About 24 nurses, with one or two members of their respective committees, availed themselves of his kind invitation. They were met at the station at Tunbridge Wells, and were driven in brakes through the park to the Castle, where they received a warm welcome from Lady Henry Nevill and the Hon. Mrs. Egerton. An enjoyable afternoon was spent. Tea was served in a tent in the park, after which an address was given by Miss Amy Hughes, general superintendent of Queen Victoria's Jubilee Institute, which was listened to with great interest. Before the nurses separated they gave three hearty cheers for Lady Henry Nevill and others who had helped to make the outing a success.

### A SUCCESSFUL POUND DAY.

Tuesday, July 10th, was advertised as the date appointed for the third Pound Day of the Kingsbridge Cottage Hospital, and an appeal was made to double the 400 lbs. sent last year. On Sunday evening, the 8th, the first gift came from a flower service. Both the matron and nurse were busy up to 10 o'clock arranging the flowers for the wards. Post, carriers, boys, girls, men, and women, gentle and simple, continued to bring pounds all day Monday, Tuesday, and Wednesday. At seven in the evening of Tuesday the rain of parcels seemed to have quieted down, when one zealous gentleman came with the results of his collections from many outlying places, to the number of 900 lbs. When the contents of the three carts had been emptied on the floor in the common room it was impossible to stand except on bundles of groceries. It looked like a San Francisco grocery store—after the shock. The matron and nurse worked until nearly twelve that night to pack the pounds into heaps, to be put away the next day. There was a pound of almost everything, from coals to writing paper. Vegetables, butter, meat, coppers, sovereigns, cheques, soap, candles, chocolate, and matches were also sent. One boy who had been a patient under treatment for more than twelve months and was cured, had collected £1, although he had left the hospital nearly a year; and the school children sent lbs. of coppers. In money alone over £10 10s. was given; and the number of separate pounds amounted to 1,876.

### BELLEVUE HOSPITAL AND THE TRAINING SCHOOL.

In consequence of the representation of Dr. J. W. Brannan, President of the Board of Trustees of Bellevue and Allied Hospitals, New York, the Board of Estimate and Apportionment have authorised an issue of 628,000 dollars in corporate stock for the construction and equipment of a training school for nurses connected with the hospital. The Board were assured by Dr. Brannan that unless this action was taken at once the support of private interests which have been maintaining the present training school would be withdrawn, and Bellevue would find itself without nurses. The new building will be erected on a site south of the hospital, covering most of the block in First Avenue, between Twenty-fifth and Twenty-sixth Streets.

### SHORT ITEMS.

The Holborn Guardians have complimented two of the nurses at the infirmary—Miss A. D. Bond and Miss Judge—on their services to the injured at the recent tramway accident at Highgate. Miss Bond, by holding a ruptured artery till one of the sufferers was removed to the infirmary, saved his life.—Miss Leah Agnes Halsall and Miss Mary Isabella Gillespie, nurses at Crumpsall Infirmary, successfully passed the June examination of the Central Midwives Board. They attended the course of lectures at St. Mary's Hospital, Manchester, but received their practical training in the maternity ward of Crumpsall Infirmary.

### The Nursing Outlook.

"From magnanimity, all fears above;
From nobler recompense, above applause,
Which owes to man's short outlook all its charm."

### QUEEN VICTORIA'S NURSES INSTITUTE.

Last week we called attention to the organisation and development of the Colonial Nursing Association which provides trained nurses for English workers throughout the British dominions beyond the seas. It cannot fail to be interesting, by way of contrast, to indicate the principal features of the work done for workers in their own homes through district nurses under the auspices of Queen Victoria's Jubilee Institute. The Council of this Institute are responsible for the arrangements which are necessary for the training of Queen's nurses, as well as for the general work in connection with district nursing throughout the country. The purpose is to provide well trained nurses to minister to the sick poor in their own homes under the influence and direction of an experienced general superintendent strong enough to secure efficiency throughout the system.

Founded in 1887 this institute has accomplished much in less than twenty years. Satisfactory progress has been made in all departments of the Institute's work, and the growth in the number of nurses available for district nursing, and their increased efficiency, are most encouraging and welcome. The Council have had the wisdom and good fortune to secure the services of Dr. Cullingworth as their representative on the Central Midwives Board, and they have raised the minimum training for village nurses from six to nine months. Seeing that the larger number of county associations have already voluntarily adopted a minimum standard of twelve months' training, the minimum for village nurses is likely to be further raised. After a great deal of consideration and much discussion, it has been determined as an essential qualification for all Queen's nurses that they shall have had three years' training in a recognised hospital. These changes prove that the management of the Institute is progressive, and we have little doubt that this spirit will be still further emphasised by the promotion of Miss Hughes from the office of superintendent of county nursing to be general superintendent of the Institute. Miss Amy Hughes has had an unusually large experience, and her general knowledge of nursing work is good and reliable.

In our issue of October 21 last we dealt with the organisation of nursing by counties. The experience of the Institute shows that the annual conference of representatives of the county nursing associations becomes more valuable each year both to the Institute and to the associations themselves. Another conference, that of the affiliated associations in London which is to be held in future every six months for the discussion of subjects of common interest and importance to the associations and the Institute, is highly spoken of by the Council. Through this latter conference steps are to be taken to secure a more general co-operation between the district nurses and the out-patient departments of hospitals. Every hospital is to be supplied in future with a list of the affiliated district nursing homes in London and its neighbourhood, so that directly the services of a Queen's nurse are desired they can be made available. Another general question of considerable interest and importance is that of school nursing. We have previously explained in this column the position of this question in the metropolis and the supply of nurses by the education authorities. The Queen's Institute maintains with some reason that the most economical and efficient arrangement for securing the services of qualified and experienced nurses in the schools is for the education authority to allow the Queen's nurses to undertake the work. It is maintained that school nursing is often of a monotonous and trivial character, which deters highly trained nurses from undertaking it as their sole employment, so that unless Queen's nurses are engaged the tendency must be to create a special class of nurse out of touch with general nursing experience. This monotony is not felt when combined with general district nursing, and the Queen's nurse is already familiar with many, or possibly the majority, of the children's parents and their homes. A departmental committee of the Board of Education is now taking evidence on the question, and it is probable that the Queen's Institute may exercise a material influence on its decision, and that it may be called upon to provide a staff of nurses for school nursing throughout London.

Anyone who studies the report of the Institute for the current year must be struck with the value of the work, and by the evidence that report affords of the energy with which it is at present conducted. The keynote of success has been struck too in the endeavour to raise the standard of Queen's nurses by inculcating a knowledge of the influence for good vested in them. Queen's nurses are becoming a social power for the improvement and education of those among whom they work. The consciousness of this is growing in the minds of the nurses themselves, with the result that they are exhibiting an increasing desire to equip themselves to carry out the higher ideals of district nursing. In the United Kingdom there are 1,260 Queen's nurses on the roll, and the number is continuously increasing. The Institute does a most excellent work on intelligent lines, and deserves all the monetary support which the most generous and capable are able to extend towards it.

### The Care and Nursing of the Insane.

By Percy J. Baily, M.B., C.M.Edin., Medical Superintendent of Hanwell Asylum.

### I.—ANATOMY AND PHYSIOLOGY.

(*Continued from page* 203.)

II. *The formation of the Chest Cavity.*—The lungs are contained within an air-tight box which we call the thorax. For our present purpose we may neglect the other contents of this box and say that it is entirely filled by the elastic lungs. Now the only means by which air can enter this box is through the air passages, which, however, communicate not directly with the interior of the box itself, *but with the lungs which it contains*; in other words, if air enters the box at all, it must do so by entering and distending the lungs.

The walls of the thorax are composed partly of muscle and partly of bone and cartilage, consisting, as already explained, of the ribs, the breast-bone, and costal cartilages, together with the muscles which clothe these bones and lie between them, while at the back of the cavity there are the twelve dorsal or thoracic vertebræ. The roof of the box through which passes the trachea is filled in by the tissues (muscles, fat, etc.) of the root of the neck, whilst its floor is composed entirely of the muscular diaphragm. The reason for the existence of all these arrangements we shall see presently when we come to deal with the *Mechanism of Respiration.*

III. *The Act of Respiration.*—This consists of three distinct phases, which follow each other in regular rythmical order. These phases are (*a*) the taking of air into the lungs or *Inspiration* followed immediately by (*b*) the expulsion of air from the lungs, or *Expiration*; and then there is (*c*) a *short pause*; after which inspiration is again repeated, to be followed in turn by expiration, and again a pause, and so on. In an adult at rest this compound respiratory act is repeated about 15 to 18 times in a minute. The larger the chest capacity of the individual the less frequently does he respire, and so in men the number of respirations per minute is usually rather less than in women. In children, for the same reason, the number is higher, being on an average from 20 to 25 per minute. The rate of respiration is increased by any muscular exertion, and various emotional states also greatly affect it. Both the rate and manner of breathing may also be affected by various diseases.

IV. *The Mechanism of Respiration.*—The act of inspiration depends upon the fact that the *cavity of the thorax is capable of enlargement* so that its total capacity may be increased, whereas expiration is dependent upon a *diminution in the size of the cavity*, and in addition the *elasticity of the lungs.* Now when the cavity is enlarged air must pass into it, since, as we have already seen, we are living at the bottom of an ocean of air which presses down equally upon every part of our body. The means whereby the capacity of the cavity of the chest is increased are:—(1) The Diaphragm.—This forms the muscular floor of the chest. When it is at rest it is arched upwards, forming a sort of dome-shaped roof to the abdominal cavity. The central part of the diaphragm is fibrous or tendinous, while the muscular contractile part is arranged around this, and is attached to the lower ribs, to the upper lumbar vertebræ, and to the lowest portion of the sternum. Hence when this muscular portion contracts or shortens the central fibrous part is drawn downwards towards the abdomen, and the vertical dimension of the chest is increased. (2) The Ribs.—Each pair of ribs forms, with the cortal cartilages and the breast-bone, a hoop. These hoops are not set horizontally, but obliquely—that is to say, they occupy the same position relatively to the earth when we are standing upright, as a hoop which is resting against a wall, and not as one which is lying upon the ground. The ribs are jointed behind to the dorsal or thoracic vertebræ. This is the highest portion of the hoop, and if we continue our simile would correspond to that portion of the hoop which rests against the wall. Now by the action of certain muscles, the muscles of inspiration, as they are called, we are enabled to move our ribs in such a manner as to raise up the lower border of the hoop. During this movement the breast-bone is thrown forward, or further away from the spinal column, and so the horizontal dimension of the thorax is increased from before backwards. But by the same muscular action which raises the ribs they are also thrown outwards, so that the horizontal dimension of the thorax is also increased from side to side. As a consequence of the enlargement of the chest cavity in these directions—namely, vertically through the depression of the diaphragm and from before backwards and from side to side—by the lifting up of the ribs, air rushes in to fill up the increased space. We already know that the only way it can get in is through the air passages into the air sacs of the lungs which it distends and stretches.

At the end of the act of inspiration the muscles which have been concerned in enlarging the cavity of the chest relax, and as a consequence the diaphragm again becomes arched up into the chest by the pressure of the abdominal contents below it, and the ribs fall, partly by their own weight and partly by muscular action—the muscles of expiration. The elastic tissue of the lungs then comes into play and drives out some (not by any means all) of the air contained in the air sacs.

During ordinary normal respiration only a very small proportion of the total amount of air contained in the lungs is changed. At the end of an ordinary inspiration an average-sized chest will contain about 220 or 230 cubic inches of air; during expiration only the odd 20 or 30 cubic inches are expelled, leaving some 200 cubic inches still in the lungs. This 20 or 30 cubic inches of air which is inspired and expired during every respiratory act is called the *Tidal Air.* If we take a very full breath—a forced inspiration—so as to expand the chest to its greatest possible capacity we can draw in about 100 cubic inches more, and this extra amount is called the *Complimental Air.* In the same manner it is possible to force out about

100 cubic inches of air beyond the tidal air by making a forced expiration, and this air is called the *Reserve Air*. But even at the end of the deepest possible expiration there still remain in the lungs about 100 cubic inches of air, which it is quite impossible to force out, and this is called the *Residual Air*. The fresh tidal air which is taken into the lungs at every inspiration mixes with the air (reserve and residual) already contained within the lungs by diffusion, and so the carbonic acid which is continually being poured by the blood into the air in the air cells is carried away, and the oxygen which is taken from it by the blood is replaced, and a certain standard of purity is maintained.

V. The *Respiratory Function of the Blood.*—The blood which is brought to the lungs by the pulmonary artery is, as we already know, venous blood. The sole object of its passage through the pulmonary capillaries is that it may gather up oxygen in which it is deficient and get rid of carbonic acid with which it is surcharged. These gases, in common with all other gases, have the physical property of being able to pass through animal membranes, and therefore they are enabled to pass through the delicate membrane which constitutes the walls of the minute capillaries and of the air cells of the lungs. Now when oxygen enters the blood it at once combines with the hæmoglobin which, as we know, is the material which gives their red colour to the red blood corpuscles, forming what is called oxy-hæmoglobin, and at the same time it converts the dull red or purple colour of venous blood into the bright scarlet of arterial blood. The carbonic acid of venous blood is not carried by the corpuscles, but is dissolved in the plasma; from this it readily passes outwards into the air cells. During its passage through the systemic capillaries the reverse of these changes takes place—that is to say, the blood corpuscles lose oxygen (the oxy-hæmoglobin becoming again reduced to hæmoglobin), which passes out of the blood into the lymph which bathes the individual tissue elements, and carbonic acid passes from the lymph into the plasma of the blood.

VI. *Differences between Inspired and Expired Air.*—The air which leaves the lungs at every expiration differs from fresh air in many respects, and it cannot be too forcibly stated that air which has once passed through the lungs is no longer fit for respiration, and should never again be inhaled. Expired air contains slightly less oxygen than fresh air, and vastly (about 100 times) more carbonic acid. It also contains much more moisture. It is also hotter than the surrounding atmosphere. As the air passes out of the lungs through the air passages and mouth it carries with it some minute particles of organic matter. This may consist in part of some of the superficial cells from the lining membrane of the air passages themselves, but is no doubt chiefly composed of microscopic particles of food which cling to the teeth, gums, and walls of the mouth and pharynx at every meal. This organic matter which is thus expelled with every respiratory act floats about in the air, where it undergoes putrefaction, and produces the close, stuffy smell which is always found in badly ventilated rooms, churches, theatres, etc., which are occupied by human beings. When taken into the lungs it acts as a poison, and is sure, sooner or later, to give rise to ill-health.

### The Nurses' Clinic.

### VARICOSE VEINS.

By "varicose veins" we understand an enlarged and irregular condition of the veins usually in the lower extremities; but veins may be varicose in any part of the body. They are caused by interference with the complete return of the blood to the heart; therefore anything that impedes the circulation in that direction must be removed, and those suffering from, or likely to suffer from, varicose veins in the legs should be careful not to wear garters or any tight band at the knee.

Varicose veins are more often to be found among people who have a great deal of standing or walking to do. Therefore it is essentially a complaint affecting nurses. I make a great point of nurses resting whenever possible, and I again repeat the necessity for this from the very beginning of a nurse's career. It is usually after a few years that nurses suffer from varicose veins, and many have to give up work entirely owing to this trouble. They are most painful, and give rise to a feeling of intense lassitude and heaviness of the lower limbs, and what may be described as a "tired pain." The feet and legs become swollen, and in the acute stage the skin may become irritated and eczema develop, which frequently results later in the formation of an ulcer.

The hæmorrhage from a vein lying in the base of an ulcer may be so serious as to cause death if not stopped at once. The leg should be well raised and a hard pad, or, better still, a coin wrapped in linen, applied at the seat of the bleeding, and the leg firmly bandaged from the toes upwards and kept in a recumbent position. On the first appearance of these troublesome veins an elastic stocking, crêpe bandage, or some equally adequate support should be worn at once. These should be applied before getting out of bed in the morning, and the end of the bed raised during the night.

Those troubled in this way usually suffer in health, and require iron tonics and plenty of fresh air. The bowels are generally constipated and must be kept regular, preferably by the use of fruit, green vegetables, and a tumbler of cold water taken the first thing in the morning.

### To Nurses.

We invite contributions from any of our readers, and shall be glad to pay for "Notes on News from the Nursing World," "Incidents in a Nurse's Life," or for articles describing nursing experiences at home or abroad dealing with any nursing question from an original point of view, according to length. The minimum payment is 5s. Contributions on topical subjects are specially welcome. Notices of appointments, letters, entertainments, presentations, and deaths are not paid for, but we are always glad to receive them. All rejected manuscripts are returned in due course, and all payments for manuscripts used are made as early as possible after the beginning of each quarter.

### Incidents in a Nurse's Life.

### AN EXPERIENCE IN SWITZERLAND.

Early in May I had the good fortune to be sent to Lucerne to a patient suffering from pneumonia which soon resolved itself into pleurisy with effusion.

Just before my arrival an old woman sent in by the Swiss doctor for the purpose of cupping had extracted about a pint of blood. As the patient was a man of full habit he was much relieved by this treatment, but when the doctor told me that he treated all his pneumonia cases in similar fashion, I felt bound to say that we did not follow on those lines in England. His remark was that he had no doubt our medical men would come back again to what I thought medieval treatment before many years had gone by.

In spite of fomentations, blisters, and iodine, the affected side did not clear up. So at last it was suggested that the air of Lucerne was too enervating and a stay higher up in the mountains was advised. During the month I was in Lucerne the weather was variable—more extreme in its way than is experienced in England. We had six days' continuous rain, then about ten days of glorious sunshine, followed by another wet period during which the clouds never lifted, so that the range of mountains across the lake were simply invisible. However, I made the most of every opportunity, and went by some of the many steamboats for an excursion when I was able. The scenery is grand in this land of Alp and glacier, mountain and flood; and the spring tints and fruit blossom weie then to be seen in perfection.

As soon as the patient was sufficiently recovered we set out by way of Berne, the Lake of Thun, and Spiez, for Weissenburg-Bad, a "curhaus" in the Sunnenthal Valley, in the Bernese Oberland, which is about 3,000 feet above sea-level. This proved to be a sanatorium built in a ravine, through which a road had been engineered along the rocky side of a mountain, through a pine forest, and where we were surprised to find the temperature higher than in Lucerne. A resident doctor was in charge, who promptly ordered the patient to bed—although his temperature was normal—and kept him there for three weeks.

During the third week sun-baths were ordered daily, which meant that the affected side was bared and exposed to the sun for two hours at a time. The fluid disappeared gradually, and after staying about three weeks longer and spending the greater part of the time in the open air, the recovery was considered complete. I must not omit to mention the mineral water of the place which was given to all the patients in varying quantities and was supposed to be a prominent feature in the cure. It came warm out of the ground and was conveyed through pine-wood pipes to the establishment and was further heated before being dispensed all round.

Most of the visitors at this sanatorium were there for chest troubles of some sort, though a casual observer would scarcely have believed it. Still a few hollow-eyed men in the prime of life told a plain story for those who could read aright, and one felt that this remote corner beneath the shadow of the Alps was indeed a resting-place for the weary where they might regain health and strength and start out afresh after a pause, invigorated and refreshed for the battle of life.

### The Victorian Order of Nurses in Vancouver.

Last year a notice appeared in our columns of the launching of the medical missionary ship *Columbia*, and it is pleasant now to be able to record the great success of that Mission to the logging camps off Vancouver Island, at the extreme west coast of Canada. Numerous services have been held on the boat, camps have been visited, and the sick or injured carried to the hospital at Rock Bay. The Mission is now almost self-supporting. The accompanying illustration gives a fair idea of the woody surroundings and of the building itself, which is of the ready-made portable house type, shipped from Vancouver, and after a sea journey of 150 miles set up at Rock Bay, a village consisting of an hotel, a shop, and a large logging camp.

The hospital was furnished by a grant from the "Minto" fund, and two Victorian Order nurses are supplied. The first to take charge was Miss Jean Sutherland, a graduate of Galt Hospital, E. Canada, who afterwards held the post of head nurse at Fort William, where she was much beloved.

It was with much zeal and spirit that she undertook what was indeed hard outpost work, and for some months she laboured single-handed. In November she was joined by Miss Franklin, an English graduate nurse from Birmingham. The record from July-December 1905 runs as follows: Total number of cases, 63; medical, 14; surgical, 49; number of operations, 33; number of hospital days, 890; number of last offices, 2; out-door patients, 147; beds, 10; nurses, 1. Not a year has passed since the hospital was opened, and now her fellow-workers mourn the passing away of one more nursing sister, who truly died at the post of duty. Within five hours, after a sudden breakdown in the midst of her work, Miss Sutherland was at death's door with acute meningitis, followed by peritonitis, only once recovering consciousness sufficiently to give utterance to her last wishes. She died at the end of four days, during which time she was carefully treated by the Mission doctor, as well as by one from the mainland, and tenderly nursed by her assistant and another nurse, hastily summoned from a distance. Her coffin was hewn out of timber from the adjacent

Rock Bay Hospital, British Columbia.

camp by affectionate hands, and, draped with the Union Jack, was shipped to Vancouver, where the last offices were rendered by the Victorian Order of Nurses stationed there. A funeral service was held, and the remains, attired in the uniform of the Order, were sent by train to the mourning relatives in Ontario.

The strong feeling called forth by this sad event from the rough men among whom Miss Sutherland has laboured, proves how valuable is the work of a missionary nurse, not only in ministering to the bodily needs, but also in softening and developing the higher instincts of those who, through absence from home and religious influences, have often become hardened, even degraded. The *Columbia,* besides having a cabin fitted for holding services, is fitted up as a marine ambulance, with two hospital cots, dispensary, operating table, splints, dressings, and instruments. The superintendent (Rev. John Antle) acts as captain of the boat, which is worked with gasoline, and Dr. W. A. R. Hulton combines engineering with his medical work, which much lessens the cost of maintenance.

The Victorian Order have now a Home in Vancouver, opened in January last, where nurses are sent for training in district work, on the same lines as at the Training Homes in connection with the Queen's Jubilee Institute. Miss Cruikshank, a graduate of Providence Hospital, Rhode Island, is the superintendent. As in England, no patients are attended who are able to pay for the services of a private trained nurse, but all are urged to contribute a small sum, according to their means. An average of 120-150 visits monthly are paid, which, considering the very wide area covered, is good. Only maternity cases are attended at night, and each nurse is expected to have had obstetrical, in addition to medical and surgical, training before commencing her four months' district work. At the end of this period she is appointed to a district of her own or to one of the cottage hospitals, which form a leading feature of the work in this sparsely populated colony. The hours of duty are 8 A.M. to 1 P.M., 4 P.M. to 6.30 P.M. Although cases of extreme destitution are never met with in this colony, yet there are many people, especially those who have recently emigrated, who are in great need of skilful nursing, for which they cannot afford to pay the ordinary fees, amounting to £3 or £3 10s. per week.

### Central Midwives Board.

### REMOVAL OF NAMES FROM THE ROLL.

THE Central Midwives Board met on Thursday, July 12, to consider the hearing of certain charges against eighteen certified midwives. There were present Dr. Champneys (in the chair), Dr. Dakin, Mr. Fordham, Mrs. Latter, Miss R. Paget, Sir William Sinclair, and Mr. Parker Young.

The first case heard was that of Caroline Spiers, charged with persistently breaking Rules E. 2, E. 3, E. 7, E. 19 (*a*). The woman had expressed her willingness to have her name removed from the roll, and her certificate was accordingly cancelled.

On the statutory declaration of Dr. Mary Smith, Inspector of Midwives, Manchester, Sarah Maria Clerke was charged with having disobeyed Rule E. 17 (*c*). The Board decided to severely censure the midwife and to request the local supervising authority to report on her work in three months' time.

Susan Hasler, on the evidence of Dr. Bennett, Inspector of Midwives for the London County Council, was charged with persistently disobeying Rules E. 1, E. 2, E. 19 (*a*). It was decided to remove her name from the roll.

Sarah Uren was charged with disobeying Rules E. 2 and E. 19 (*a*), and with having attended a confinement when suspended by the local supervising authority (Devonport). It was decided to remove her name from the roll.

A long case was heard against Ita Feldmann, midwife, on the evidence of Mr. Swinson, Inspector for General Purposes (London County Council). The charges were that Mrs. Feldmann had on two different occasions employed her husband, an unqualified person, to discharge the duties of a midwife, for which she was employed, and on a third occasion had called him in in the place of a medical practitioner. Mrs. Feldmann was defended by a solicitor. It was decided to remove her name from the roll.

The case of Eliza Willis, employed by the Wingfield Nursing Fund, was then heard. She was defended by a solicitor, the chief charges against her being that she had attended a tuberculous abscess case and then attended as a midwife without using disinfectants, had not called in a doctor when the temperature rose above 100.4° F., and disobeyed Rules E. 1, E. 2. The first charge she met by stating that she ceased to attend the case as soon as she was told it was infectious, and had disinfected herself; she denied that the temperature rose as stated, and she produced her dresses and a full bag of antiseptics and appliances, evidently well used. The Board decided to merely caution her.

Phoebe Hoy, midwife, whose ignorance had apparently been the cause of a patient's death, was cautioned, Dr. Thresh, Medical Officer of Health (Essex), and his assistant, Dr. Stevenson, stating she seemed an intelligent person and would probably improve. The Board requested a report in three months' time.

Ellen Buck was removed from the roll, it being proved, on the evidence of Miss Bernard Boyce, Inspector of Midwives for the Norfolk County Council, that she had attended several confinements while under suspension and had persistently disobeyed the rules of the Board.

Dr. Gregg, Inspector of Midwives for South Staffordshire, gave evidence against Elizabeth Campbell and Hannah Porter. It was decided to remove the name of the former from the roll, it being proved she disobeyed Rules E. 2, E. 19 (*a*), had neglected to report a still-birth, and was entirely unfit for her work. The certificate of Hannah Porter was likewise cancelled, the evidence against her going to show she had disobeyed Rules E. 1, E. 15, E. 17.

Mary Ann McGrath was removed from the roll, on the statutory declaration of Dr. James Little (Supervising Officer of the Maryport Urban District Council) for disobedience of Rules E. 1, E. 2, E. 19 (*a*).

Emily Smith (Derby) was severely censured for disobeying Rules E. 6, E. 15 (*b*), E. 18 (1).

Margaret Ellen Manns (West Riding) was charged with disobeying Rules E. 5, E. 15, E. 17 (*b*), E. 17 (*c*). E. 19 (*b*). The death of one patient had resulted and the serious illness of another. It was decided to remove her name from the roll. Alice Hilton was removed from the roll, for disobedience to Rules E. 5, E. 17 (*c*), E. 19 (*b*), on several occasions. Evidence was provided by Miss Edith Wright, Inspector of Midwives, Prestwich, in this case and also in the case of Nanny Lord, likewise removed, for disobeying persistently Rules E. 1, E. 2.

Sarah Patrick (Birmingham) charged with drunkenness, and disobedience to several of the rules, was removed from the roll.

It was decided to take no further action in the cases of Elizabeth Rogers and Mary Jane Blackler.

### Wants and Workers.

A DISTRICT NURSE would be grateful to anyone who has a spinal carriage to give away to a poor girl of twenty suffering from chorea, who is unable to get out of doors, as she cannot sit up at all. Her parents are very poor. The nurse would willingly pay carriage if sent addressed to her at Elm Villa, Warsash (Swanwick Station), Hants.

### General Lying-in Hospital.

SHOW OF BABIES.

On Thursday last week a successful show was held at the General Lying-in Hospital, York Road, for all babies of patients at the hospital born in 1906. Nearly 200 babies were brought by their mothers, and as only a week's notice had been given of the show, the general fat healthy condition of the babies did the mothers great credit, and bore excellent testimony to the teaching instilled into them during their stay in hospital. The judging was undertaken by Mrs. Messenger, Dr. Lionel Smith, and Dr. D. Smith, and could have been no easy task. The mothers, as they entered the grounds were divided into classes according to the months when their babies were born, by the aid of a burly policeman who took the liveliest interest in the whole proceedings. They were then seated in "monthly" groups under an awning, where later a good tea was provided. It was a little noisy at times with so many vigorous babies, still they did not all cry at once and many of them looked the picture of placid content. There was a good number of prizes for the healthiest and best-kept infants, consisting of babies' clothing, six garments being allotted to each month. Lady Victoria Lambton gave away the prizes, a bunch of cornflowers accompanying each gift. Inside the hospital there was much pleasant excitement, as it was thrown open to inspection, and all the babies were made very smart with pink or blue ribbons, pink for girls and blue for boys. The latest arrival, only an hour old, came in for much admiration, being a remarkably fine boy, scaling 9 lb. 10 oz. Great was the importance of the adopted baby of the hospital "Tim" (a girl, by the way) who had weighed only 2 lb. at birth and used to be rolled up in cotton-wool and laid on the window-sill in the sun. In spite of her bad start in life she throve on this treatment, and at thirteen months is quite an average child and the pet of everyone—matron, doctors, sisters, and nurses. A new storey has been added to the hospital, which provides much needed extra accommodation for nurses and the domestic staff. Want of space compelled the adoption of the cubicle system and made it necessary to share a window between two cubicles. No pains have been spared to make the nurses' quarters as comfortable as circumstances would allow.

### Presentations.

Borough of Widnes.—On Wednesday last week there was an interesting presentation, under the auspices of the Borough of Widnes Education Committee to Nurse M. Starbuck, of a handsome occasional chair, as a small token of regard for the excellent lectures which she has given on Home Nursing, bearing an inscription on a silver plate. Mr. J. H. Danby, Director and Secretary of Education, presided, and Mr. W. H. Sutton Timmis, who made the presentation, mentioned that Widnes was the first borough to attach a Queen's nurse to an education staff. Nurse Starbuck, in acknowledging the gift, said that if she had not had a very intelligent class she could not have obtained the satisfactory results which had been alluded to.

Stratton Cottage Hospital.—Miss E. Phillips was presented upon leaving Stratton Cottage Hospital with a travelling clock with the inscription: "Presented to Sister Phillips by the members of the committee of the Stratton Cottage Hospital, upon her leaving the hospital, as a token of personal regard and in recognition of the excellent services rendered by her to that institution during the five years she had acted as matron." The Ladies' Committee presented a handsomely fitted nurse's bag. Miss Phillips is leaving to take up private work in Edinburgh.

### Everybody's Opinion.

TWO YEARS' SYSTEM OF TRAINING.

"X." is requested to send name and address, as no card was enclosed with the letter.

AUSTRALIAN NURSES IN SOUTH AFRICA.

Miss E. Glover, Acting Hon. Secretary of the Royal Victoria Trained Nurses' Association, writes: Great indignation prevails in Victoria over Miss H. Kenealy's contributions to the *British Journal of Nursing*. She has met a few colonial nurses in South Africa who failed to come up to her standard, and unjustly and hastily she sums up the whole sisterhood as lacking in every good quality. Some of the greatest failures I have met in my nursing career in this country have been English trained, but the people on this side have been too courteous to judge the whole by the few.

THE EXAMINATION OF THE CENTRAL MIDWIVES BOARD.

Mrs Herbert Thompson, Hon. Secretary of the Cardiff branch of Queen Victoria's Jubilee Institute, writes: Commenting on the note in your issue of July 7 with regard to the last Central Midwives Board examination, it is true that on this occasion three out of four of the candidates trained by this institute failed, although on previous occasions we have been very successful. It will perhaps prevent misunderstanding on the part of the public if it is made clear that the candidates were not Queen's nurses but had only been under our institute for the three months' midwifery training.

INCIDENTS IN A NURSE'S LIFE.

"Edinburgh Nurse" writes: I have just read the article "In a Private Asylum," and cannot pass over the references the writer makes to "ordinary nurses" and "attendants." She speaks of one of the latter, who, by her own showing, had been for some years a devoted companion and capable nurse, as "attendant," and talks of calling up the "ordinary nurses." Why allude to them in this slighting manner, when they are doing such noble work, which requires years of experience, for there is no disease that needs more skilful nursing than mental disease? Surely the time has come to drop the use of the word "attendant" in respect of nurses who devote their lives to the care of the insane. But this work requires sympathy, of which the writer of the article seems to have very little, judging by the way she talks of the "lunatics," and thinks that after sixteen months she did well to give up the work. I have had nine years' experience in mental nursing, and I find it most interesting; also I feel that I am doing a little good in the world by trying to cheer and brighten the mentally afflicted.

THE PENSION FUND REPORT.

"One who has Really Benefited and is Grateful" writes: On reading your report of the Pension Fund I wondered if my experience as a member of it would be of any value to the younger nurses in encouraging them to join. Though not expecting to require its assistance, I thought all nurses should join, not only to increase its funds, but because none can be sure of continued prosperity no matter how safe the future may seem to them. Now I find myself growing old, and owing to advancing years and failing strength work is difficult to find and to do. In my case, what makes such a time more sad, I suddenly find that the means to live independently have vanished, and the small annuity, with but a trifle more, is all that I have to call my own. Having through life had to battle with continued ill-health, I have no need to tell any nurse that much could not be saved. The Pension Fund never had to assist me previously, but I was kindly allowed time when I had to recruit my strength or wait for work. Now, fortunately, the annuity can be lying by if I remain well, and so increase in readiness for a time when I must give up altogether. Having home and friends for many years I did not see the great value of the Sick Fund, but bitterly did I regret the last few years that I had never joined. Nurses are not well paid, if we are to judge from the salaries offered in so many of the advertisements. From the numbers joining I should say that, in the majority of cases, they will be still worse paid in the future. Luxury and extravagance are too much on the increase in every class to allow of greater salaries being given to either hospital or private nurses. Let nurses co-operate universally now that they have such a fund at their disposal, and show not only their gratitude to those who founded it, but also the practical side to their character, added to a determination never to become an object of charity or a burden to those unfortunate ones whom fate has made their nearest of kin.

---

### ARE NURSES UNDERPAID?

"Matron" writes: I think that nurses cannot be sufficiently grateful to Mr. Pollitt for having brought forward the subject of their inadequate payment. We are most of us in the unfortunate position of being unable to speak for ourselves lest from us "who have not shall be taken away, even that we have"; but a glance through the advertisement columns of our valued paper The Hospital, tells its own tale of the pitiful salaries offered to trained nurses, and the rush for posts of only very moderate financial value, corroborates it. After ten or fifteen years' hard work and varied experience a nurse may think herself lucky to secure a matronship of £35, or a district at £30. Why are nurses so cheap? I venture to think that one reason is the great influx into the nursing ranks of those who would otherwise be domestic servants (now almost extinct) yet who as nurses are content to accept a servant's wages. I agree with Mr. Pollitt that the flower of the nursing profession is drawn from the educated classes, but a gentlewoman can hardly live and save for the future on £30 a year. As a member of the Royal National Pension Fund (second thousand), I look forward to the yet far-distant day when I shall receive my pension (£22 10s.), but though it will help, it will not keep me, and larger premiums are out of the question with a salary of £35 a year!

"Nurse E" writes: I do not think that Mr. Pollitt's deduction from data supplied by the reports of the Pension Fund that a nurse can only save enough to assure 8s. a week is conclusive. I am one of the 627 annuitants of the Pension Fund and at present receive a very small annuity; but in four years' time I shall be in receipt of a pension of £78 per annum, and no doubt several other annuitants are similarly circumstanced. Then again, many nurses (like myself) have set aside a substantial sum to provide a home after they have given up nursing and not sunk all their savings in annuities. I know three nurses intimately, one of whom invested her savings in house property which brings her in a good but variable income. The second invested hers in deferred annuities and will have about £100 per annum. The third allowed her mother, who was paralysed, 15s. a week for over seventeen years. This last may not have been able to assure more than 8s. a week, but that was not because she was underpaid. These three nurses are women of about fifty years of age, they are nursing on their own account, and their health does not appear to have suffered from their thrifty habits. Why not ascertain what annuities the second thousand nurses of the Pension Fund will obtain, and perhaps a more correct solution of the amount that nurses can save will be forthcoming?

"Nil Desperandum" writes: May I be allowed to say a few words, as suggested by Mr. Pollitt, not as to whether nurses are underpaid, but as to whether they could have done better in the way of making provision for old age? Speaking for myself, I began nursing eighteen years ago, and, with the exception of two years' illness, have been engaged in nursing for that period. Since the completion of my training and excluding the two years mentioned above, my earnings have averaged £25 per annum, together with material for indoor uniform. Out of this I have contributed £10 per annum towards the support of an invalid mother; and since 1898 I have belonged to the Pension Fund, and if I can continue to pay £9 14s. per annum for a further period of sixteen years I shall be entitled to a pension of £20 per annum, or 7s. 6d. per week. From this you will see that I have not had a very large margin for holidays or amusements. Please do not think that I am complaining. I am very happy; every moment is fully occupied, as I have all my clothing to make; also I am passionately fond of walking and reading—amusements that fortunately for me cost very little—and there are countless other sources of enjoyment. "Thou shalt be served thyself by every sense of service which thou renderest." The greatest deprivation is the holiday. I have never been able to afford a real holiday. At the present moment I am very rich, as I have been receiving a salary of £35 for the past year, so I am trying to save a few pounds to furnish a cottage. Rooms would be out of the question. I went to inspect some for a friend the other day. They were so tiny and quite barely furnished, and they were £1 a week. I am looking forward to getting a cottage in the country for something under 2s. 6d. per week—that is, if they have not all been appropriated as week-end cottages by millionaires; then if I have a little money for furnishing and am able to do my own work I shall manage to fare quite luxuriously on the 5s. a week. If I find I cannot keep on until my pension is due, I shall withdraw my money, and do the best I can with it as long as it lasts. In case any of your readers may consider that I might have been doing better after so many years' work, I may say that I am not physically strong enough either for private nursing or the work of a large hospital. I am not eligible for the sick branch of the Pension Fund.

---

### NURSES OFF DUTY.

"A London Hospital Nurse" writes: We are always having the fact pointed out to us that "nursing is primarily woman's work." The writer of nearly every "text-book" on the subject begins with this valuable statement. He or she (as the case may be) lays down all the laws she can, and makes use of the time-worn maxim of woman's unfailing sympathy with the sick and suffering, especially if the sufferers should be children. The author then proceeds to enumerate the specific qualifications which an applicant should possess; if she possesses them, she should certainly be ranked amongst the angels. It is an acknowledged fact that if one becomes part of a public institution one must drop one's identity, and become part of the general machinery; more especially is this so on entering a hospital. Everything that the authorities can do to render individuals uniform is done, and should, by any chance, one individuality escape the general uniformity, she is regarded with great suspicion. Everyone not completely devoid of sense realises that it is absolutely necessary that certain rules should be made and kept, but surely when a nurse is off duty she should be allowed to assume her individuality, and to develop it as much as possible, free from all petty tyrannies! In one large London hospital it is against the rules for a nurse to have any flowers in her own room, and she is only allowed to have a certain number of ornaments about. Yet this is her private sanctum, the only place where she can ever be sure of being alone and uninterrupted, and it is a criminal offence to have a few flowers and more than two photographs on her dressing-table. In yet another hospital there is a stringent rule about how far open the window is to be kept. No provision is made for the weather, and if a nurse wishes to take a friend to her room, that being the only place where she can hope for any privacy, she has to answer an array of questions which would satisfy a Sherlock Holmes before she can obtain permission. In short, she is treated as a child who is ever ready to fall into serious mischief, and who must be well looked after; but when on duty another order prevails. She is supposed to be an educated woman, capable of looking after the people entrusted to her care. Can any sensible person reconcile these two extremes? One is constantly reading in illustrated papers of the noble buildings in which nurses are housed, but no one ever publishes a list of the petty tyrannies to which they are subjected, of the variety of food they are given to eat. Again, no one ever inquires into the way in which it is cooked. An alluring picture is presented of their life off duty, but only the surface is skimmed over, and the depths and cesspools are left untouched.

### Appointments.

[No charge is made for announcements under this head, and we are always glad to receive and publish appointments. The information, to insure accuracy, should be sent from the nurses themselves, and we cannot undertake to correct official announcements which may happen to be inaccurate. It is essential that in all cases the school of training should be given.]

British Home for Incurables, Streatham.—Miss Ellen Walker has been appointed staff nurse. She was trained at University College Hospital, London, where she has since been sister.

City of London Hospital for Diseases of the Chest.—Miss A. Stuart Cameron has been appointed Assistant Matron. She was trained at the Dumfries and Galloway Royal Infirmary. She has since been ward and theatre sister at the General Hospital, Wrexham; night sister at the Royal Infirmary, Preston; ward sister at the General Hospital, Wolverhampton, and home sister at the Royal Ophthalmic Hospital, City Road, London, E.C.

Crumpsall Infirmary, Manchester.—Miss Mary Unsworth, Miss Ellen Rylands, and Miss Sarah Ann Flilcroft have been appointed charge nurses. They were trained at Crumpsall Infirmary and recently passed their final examination.

Ellon District Epidemic Hospital, N.B.—Miss Ellen Murray has been appointed staff nurse. She was trained at the Summerfield Hospital, Aberdeen, and afterwards became assistant nurse at the Gordon Hospital. She has also done private nursing at Aberdeen.

Enfield Cottage Hospital.—Miss Elsie Hunsford has been appointed staff nurse. She was trained at the Bristol General Hospital, and has since been staff nurse at Shaftesbury Cottage Hospital.

General Lying in Hospital.—Miss Alice Park, home sister at Guy's Hospital, has been appointed matron of the General Lying-in Hospital, York Road—not the British Lying-in Hospital as erroneously stated in our issue of July 14, p. 222.

Hutton Nursing Home, Ceylon.—Miss Elizabeth Moorhead has been apointed to the private staff by the Colonial Nursing Association. She was trained at Crumpsall Infirmary, and has since been charge nurse at Park Hospital, Hither Green. She has since been engaged in private nursing at Cromer. She holds the certificate of the Central Midwives Board.

St. Mark's Hospital, City Road, London.—Miss Frances Sherriff has been appointed night sister. She was trained at St. Luke's Hospital, Halifax, has been assistant nurse at St. Martin's Hospital, City Road, and has done private nursing. She holds the certificate of the Central Midwives Board.

Swansea General Hospital.—Miss Catherine Ellen Bennett has been appointed assistant matron. She was trained at St. Bartholomew's Hospital, London, where she has since been sister. She has also been sister at the New Hospital for Women, Euston Road, London, and sister at the Royal Hants Hospital.

West Down School, Winchester.—Miss Anna M. Vibart has been appointed matron. She was trained at the Royal Devon and Exeter Hospital, and has since done private nursing for the Royal Hants County Hospital, Winchester.

Whiston Union Infirmary.—Miss Hilda Spiller has been appointed charge nurse. She was trained at Toxteth Park Infirmary, Liverpool, and Queen Charlotte's Hospital, London, and has since been charge nurse at Toxteth Park and Dewsbury Union Infirmaries.

Wolverhampton Workhouse Infirmary.—Miss Beatrice Porter has been apointed charge nurse. She was trained at Saffron Walden Hospital, and subsequently at West Ham Infirmary. She has since been charge nurse at West Ham Infirmary and Kendray Hospital, Barnsley.

### TRAVEL NOTES AND QUERIES.

By our Travel Correspondent.

Hotels in Belgium (K.M.).—We do not reply by post unless 2s. 6d. is sent—see rules. Utrecht is far removed from the Ardennes and you would not touch upon it in going there; however, if you must stop there, which will greatly increase the expense of your journey an inexpensive hotel is the "Oude Trecht." At Ypres the best hotel is in the west corner of the Grande Place. Huy is somewhat off your line, and not a place to stay in; you will see it from Namur. The best place to stay at in the Ardennes is Dinant—Hôtel Tete d'Or—the best terms 7 francs per day. Hôtel des Familles cheaper. Always ask for rooms on the third floor. You have not asked too many questions. I could have helped you better if you had been more explicit.

Holiday in Dublin (Pocket Edition).—I know of no home such as you mention; but at the following addresses you will find respectable and moderate accommodation; Mrs. McIntosh, Cabar Teidh Hotel, 107 Stephen's Green; Mrs. Evans, Auburn House, 11 Rathmines Road; Miss Fletcher, Residence House, 8 Upper Pembroke Street; Mrs. Nichols, 6 Lower Fitzwilliam Street. This last is only for governesses, so I fear it is no use for you.

Boarding House in Bognor (E. J. T.).—Thanks for letter and enclosure. I am glad to have it.

A Sea Trip in July (J. S.).—We do not answer by post unless 2s. 6d. is sent for our Convalescent Fund. You are requested to give full time for an answer to appear in these columns, and I did not receive your letter till almost the last minute. You do not tell me what you can afford, but if you want very reasonable terms you cannot do better than apply to Miss Snell, Ivy Cottage, Caerhays, near St. Austell, Cornwall—her house is by the sea. Or write to Mrs. Alan Murray, Brookside, Croyde, North Devon—also by the sea. I cannot advise you as to a sea trip because you have left it too late; all berths for that date are booked long since. Should the date of your holiday turn out to be later, write to Dr. Lunn, 5 Endsleigh Gardens, W.C., and ask for particulars of his sea trips.

Ireland for Two Weeks (Sullivan).—I fear that what you propose doing will be rather costly. At Dublin the cheapest hotel which I can recommend is Rippingale's Temperance Hotel, 2 Harcourt Street, from 7s. a day. If you can stay a week in the Vale of Ovoca write to Mrs. B. Carter, Tinnahuich Cottage, Vale of Ovoca. Her terms are from 25s. per week. At Bray try Wallis's Hotel, 12 Goldsmith Terrace, or the Station Hotel, both reasonable.

Pau for Three or Six Months (L. A. S.).—It is an expensive place, but you might write in my name on a prepaid foreign return postal card to Madame Laborde-Faisans, 26 Rue Porte Neuve, Pau Basses Pyrenees, France. I think low terms may be secured for a long stay. Yes, it is a good centre for excursions; Eaux Bonnes, Eaux Chandes, Argèles, Lourdes, and Cauterets are all within easy distance. If you decide to go there write again, and I will answer your other questions.

Rules in Regard to Correspondence for this Section.—All questioners must use a pseudonym for publication, but the communication must also bear the writer's own name and address as well, which will be regarded as confidential. All such communications to be addressed "Travel Correspondent, 28 Southampton Street, Strand." No charge will be made for inserting and answering questions in the inquiry column, and all will be answered in rotation as space permits. If an answer by letter is required, a stamped and addressed envelope must be enclosed, together with 2s. 6d., which fee will be devoted to the objects of "The Hospital" Convalescent Fund. Ten days must be allowed before an answer can be published.

### A Book and its Story.

### A VICEROY'S CONSCIENCE.*

INDIA is the scene of Mrs. Everad Cotes' clever novel, which in its impartiality, its consistency, its power of presenting all sides of its subject for the reader's consideration, possesses some important elements not usually found in books, with a purpose, written by women. It deserves intelligent and careful perusal, and is not a book to be taken up and read for the mere amusement which the dialogue and the scenes from Anglo-Indian station life, with some others from London drawing-rooms, afford; but for the active demonstration of a principle—the equality of all races, irrespective of creed or colour—to which Lord Thame, the newly-appointed Viceroy, who has only a theoretical knowledge of the subject, has pledged himself. His fitness for the position is questioned by his Chief Commissioner, whose acquaintance with Indian affairs has been long and practical, in the following *mot*: "He is a man with a violent conscience and a rather short perspective." Anyhow the strength of his convictions leads him to overrule the finding of the District Court and to order a new trial for the case of an English private soldier charged with the murder of a native, which leads to a sentence of death, frustrated, however, at the last moment by the poisoning of the prisoner by opium, and a discovery, too late, that the murdered native is still alive, and that the evidence was a compound of falsehood and treachery, by which a man, greatly to blame and altogether a *mauvais sujet*, is yet not guilty of the capital crime. Interwoven with much subtlety is the human and personal thread of interest which runs gaily and discreetly, and in one case with tragic intensity, throughout the maze of events which go to make the story. The happiness of Victoria Tring, a cousin of Lord Thame, an original and charming girl, with whom he is in love, who declares that she has every appreciation for him but the desire to marry him, is bound up with this part of the drama. She stands for inspection, and comes on the scene after having just received the news of his appointment. "The hair about her forehead was damp, her eyes were dark and enthusiastic. She had the look of being blown in, and a current did seem to come with her that lifted the atmosphere of the room. They all felt it and were quickened." Congratulations have been pouring in upon Anthony Thame's mother on her son's appointment, and various opinions are expressed on the subject of a Viceroy's position, when Victoria expresses her views notably. "I should think," said Victoria Tring slowly, "that to rule an alien people must be one of the few things in which there is essential glory. To be a king among one's own is a mere accident, without, as far as I can see, any compensations, unless you count not having to catch trains. But to be imposed that way from above, over strange races, with different joys and sorrows and ambitions, whose knees really tremble, and whose eyes really look up, it is like holding a mission from the gods." Victoria has a very real sorrow in her life in a brilliant but unprincipled brother, who has mysteriously disappeared, and no efforts have availed to trace him. To her he had ever shown his charm, and had found it worth while to be at his best with her. His mother being a woman who rode in every van of progress, more given to schemes for the public advancement than those connected with home life, took the matter philosophically. She had had little to do with the education and training of her son, who had early been given into the charge of tutors and crammers; so later, when "debt and dissipation rose like phantoms where this son had been, it could not point the way he had taken." With her advanced views America was a country towards which Mrs. Tring looked optimistically. "To America she could resign her son with something like confidence. America would offer him a career, whatever he had upon his conscience; and in the relief of this conviction Mrs. Tring's head, as fair and fluffy as ever, was soon bent with its accustomed absorption over the evening paper. America, after all, had been open to him, whether he had gone there or not." Under an *alias* the lost son plays a conspicuous and sordid part in the story. Pilaghur, the Indian township where it is chiefly enacted, will not perhaps be found difficult of recognition to those who know it under another name. "Pilaghur is the capital of the province of Ghoom, which is ruled by a Chief Commissioner, under the Government of India, under the Parliament of Great Britain and Ireland, under the King. . . . . Reflecting, we may call Pilaghur two capitals. There is the Pilaghur which the ladies of the station think of, and which figures—it has a cathedral—in diocesan reports. It has a parade ground as well as a cathedral, and a station club, where are tennis courts and the English illustrated papers, and public gardens set with palms and poinsettias, while the band plays twice a week in the evenings after polo. Two or three roads lie fairly parallel in Pilaghur. . . . You could walk for nearly a mile on some of them before being lost again in the incomprehensible agriculture of Ghoom. The sun-suffused roads run up into rubbly banks on either side, and these are crowned with grey-green cactuses, many-armed and dusty. The native quarter of Pilaghur has its picturesque features. "Never is there room for the tide of life that beats through it, chaffering and calling, ox-carts pushing, water-carts trolling, vendors hawking, monkeys thriving, and Ganeshi Lal, who wires a price to London for half the seed-crops of Ghoom, looking out indifferently from his second-story window over it all. Ganeshi sits on the floor; he wears a gold-embroidered velvet cap, and the scalloped window frames him to the waist." There are a hundred such pictures. Among the Anglo-Indian circle not the least interesting figure is Miss Pearce, "who properly comes last because she had no quotable position." The table of precedence does not provide for demi-official lady doctors, having been invented before they were." Miss Pearce is a gifted and attractive woman, who has a high standard of duty, to which personal and social claims are made subordinate. Love, for the same reason, is relegated to the background of her life—or, at least, love which cannot be accepted without the sacrifice of these principles, and so stands outside the question for her. She is returning from a morning drive, and her native servant Hiria awaits her. "She passed Hiria without seeming to see her . . . without seeming either to hear her. . . . Hiria followed dissatisfied, reflecting, 'She comes always thus, walking in her sleep from the talk with Burra Sahib.' Then Miss Pearce has to listen while Hiria recounts the names of callers during her absence. "The Gunga Dass has been here again this morning, Miss Sahib. 'Oh, Ayah-ji,' he cried, 'implore the Miss Sahib to come quickly." It is his wife this time. She is fourteen and has eaten too many mangoes.' That Gunga Dass would write nothing on the slate. 'It is always cheaper,' he said, 'not to write'; and that is true talk, but shameful, for Gunga Dass has half a lakh, and seven houses in the Bazaar. Yet has he never paid we folk for curing his mother of the rheumitation last year—and she walks now as straight as a Brahminy cow, and it appears to me that neither will he now pay for his wife. If she die, he can get another." Miss Pearce smiles, but the smile was only half connected with the philosophy of Gunga Dass, and had a deeper source. "I will go this afternoon," she said. There are many pen-portraits in "Set in Authority," drawn with vivacity and accuracy, and Sir Ahmed Hossein, the Mohammedan District Judge, is among the more distinguished. Mrs. Everad Cotes' very up-to-date story of Indian life will add one more laurel to her already well-established literary reputation.

* "Set in Authority." By Mrs. Everad Cotes (Sara J. Duncan). Constable. 6s.

### Notes and Queries.

**REGULATIONS.**

**The Editor is always willing to answer in this column, without any fee, all reasonable questions, as soon as possible.**

**But the following rules must be carefully observed.**

1. **Every communication must be accompanied by the name and address of the writer.**
2. **The question must always bear upon nursing, directly or indirectly.**

**If an answer is required by letter a fee of half-a-crown must be enclosed with the note containing the inquiry.**

*Nursing Institutions at Montreux.*

(192) Are there any nursing institutes at Montreux?—*Harpenden.*

No. Possibly Dr. Stuart Tidey, Belle Rive, Montreaux, would advise you.

*South Africa.*

(193) Would a person suffering from bronchitis or asthma be benefited by residing in South Africa? Could a trained masseuse make a living there, and does the Pension Fund extend to masseuses, and is there a limit of age?—*Melberta.*

Possibly if you write to the Matron of one of the South African hospitals she will advise you both as to climate and as to the prospect of success as a masseuse. The Pension Fund is solely for hospital nurses and officials, and there is no limit as to age; only necessarily the premiums are higher for those who join when they are no longer very young.

*A Monthly Nurse's Fees.*

(194) After engaging me to attend her, a lady has written that she does not want me. What is my remedy?—*M. E. C.*

You can claim half the fee, and in your case possibly more; but probably you will not care to go to law, as the sum in question is not large.

*Anonymous.*

(195) We do not answer anonymous inquiries.—*F. G. S.*

*Droitwich.*

(196) Can you tell me of an inexpensive home at Droitwich where a nurse could go for a cure?—*District Nurse.*

If you do not wish to go to the St. John's Brine Bath Hospital, where paying patients are received for £1 2s. a week, perhaps some of our readers could help.

*Twenty-one Years of Age.*

(197) I have a friend of twenty-one desirous to become a nurse, and she desires information.—*Harrow.*

She will find all she needs in "How to Become a Nurse," published by The Scientific Press, 28 Southampton Street, Strand.

*Mental Nursing.*

(198) I wish to train as a mental nurse; can you advise me or give me the necessary information through your paper?—*B. G.*

You had better write to the asylums direct. You will find a list and all information in "How to Become a Nurse," The Scientific Press, 28 Southampton Street, Strand. Price 2s. 2d. post free.

*Scholastic.*

(199) Can you give me the names of two scholastic papers, as I want to get an appointment in a school sanatorium?—*Aberdeen.*

"Journal of Education," monthly, 3 Broadway, Ludgate Hill, E.C.; "Schoolmistress," weekly, 149 Fleet Street, E.C.

*Zenana Nursing.*

(200) Can you tell me about Zenana nursing in India, and do some steamers take nurses as stewardesses?—*Betty.*

Your question is too vague. Write to the Zenana Bible and Medical Mission, 2 Adelphi Terrace, W.C. The two Zenana Hospitals in India only employ native nurses. The Booth Steamship Company, Adelphi Terrace, W.C., employ trained nurses on board, and the Royal Mail Steamship Company, Southampton, employ nurses occasionally as stewardesses, but vacancies are exceedingly rare.

**Handbooks for Nurses.**

| | Post Free. |
|---|---|
| "How to Become a Nurse: How and Where to Train." | 2s. 4d. |
| "Nursing: its Theory and Practice." (Lewis.) ... | 3s. 6d. |
| "Nurses' Pronouncing Dictionary of Medical Terms." | 2s. 6d. |
| "Complete Handbook of Midwifery." (Watson.) ... | 6s. 4d. |
| "Preparation for Operation in Private Houses." ... | 0s. 6d. |

Of all booksellers or of The Scientific Press, Limited, 28 & 29 Southampton Street, Strand, London, W.C.

### For Reading to the Sick.

CONSOLATION.

"Art thou weary, tender heart?
 Be glad of pain;
In sorrow sweetest things will grow
 As flowers in rain.
God watches, and thou wilt have sun
When clouds their perfect work have done."

Is it raining, little flower?
 Be glad of rain.
Too much sun would wither thee.
 'Twill shine again.
The sky is very black, 'tis true,
But just behind it shines the blue."

*Anon.*

When consolation is taken from thee, do not immediately despair; but with humility and patience wait for the heavenly visitation, for God is able to give thee back again more ample consolation.—*Anon.*

Many of the world's best things have been born of affliction. The sweetest songs ever sung on earth have been called out by suffering. The richest blessings that we enjoy have come to us out of the fire. The good things we inherit from the past are the purchase of suffering and sacrifice. Our redemption comes from Gethsemane and Calvary. We get heaven through Christ's tears and blood. Whatever is richest and most valuable in life anywhere has been in the fire. Our love for one another may be strong and true in the sunny days, but it never reaches its holiest and fullest expression until pain has touched our hearts, and called out the hidden treasures of affliction.—*Dr. J. Miller.*

Be ye of good cheer, every one that is afflicted, for the Lord is preparing for you the city of God. Whatever be your sorrow, it is the token of His love; for the Man of Sorrows is our King, and the path of sorrow is the path of His Kingdom; there is none other that leadeth unto life. Your reward is sure if you are but true to yourself. Do we believe these things? Are they realities, or are they words? They are God's Word, which is a reality.

If any sincere Christian cast himself with his whole will upon the Divine Presence which dwells within him, he shall be kept safe unto the end. When did we ever set ourselves sincerely to any work according to the will of God, and fail for want of strength? It was not that strength failed the will, but that the will failed first. If we could but embrace the Divine Will with the whole love of ours, cleaving to it, and holding fast by it, we should be borne along as upon the river of the water of life.—*H. E. Manning.*

There are briars besetting every path,
 Which call for patient care
There is a cross in every lot,
 And a need for earnest prayer;
But a lowly heart that leans on Thee
 Is happy anywhere. *A. L. Waring.*

## Figures and Tables

**Figure f1:**